# Incidence and Factors Associated with Neurological Complications Following Pediatric Heart Surgery: A Retrospective Study

**DOI:** 10.3390/jcm15051721

**Published:** 2026-02-25

**Authors:** Nurdan Yılmaz, Murat Uğurlucan

**Affiliations:** 1 Department of Anesthesiology and Reanimation, Istanbul Medipol University Hospital, Istanbul 34214, Türkiye; 2 Department of Cardiovascular Surgery, Istanbul Liv Hospital, Istanbul 34485, Türkiye; muratugurlucan@yahoo.com

**Keywords:** pediatric cardiac surgery, congenital heart disease, neurological complications, cardiopulmonary bypass, postoperative outcomes, neurodevelopment

## Abstract

**Background/Objectives:** Neurological complications remain a critical source of morbidity and mortality following pediatric cardiac surgery for congenital heart disease (CHD). While advances in surgical and perioperative care have improved survival, the incidence of postoperative neurological events and their associations with perioperative characteristics remain incompletely characterized in heterogeneous pediatric populations. This study aimed to assess the incidence of postoperative neurological complications and to examine factors associated with adverse clinical outcomes in pediatric patients undergoing cardiovascular surgery. **Methods:** This retrospective, single-center study included 210 pediatric patients (<18 years) who underwent open-heart or major cardiovascular surgery with cardiopulmonary bypass at Medipol University Hospital between January 2021 and January 2022. Univariable logistic regression analyses were performed to explore associations between perioperative variables, postoperative neurological complications, and in-hospital mortality. **Results:** Of the study population, 119 patients (56.7%) were male and 91 (43.3%) were female; 117 patients (55.7%) were younger than 2 years of age. The most common procedures included ventricular septal defect repair (18.6%) and tetralogy of Fallot repair (13.3%). Postoperative neurological complications occurred in 20 patients (9.5%). Median postoperative intubation duration and intensive care unit (ICU) stay were significantly longer among patients with neurological complications (*p* < 0.001). In-hospital mortality occurred in 18 patients (8.6%). Postoperative neurological complications, reoperation, prolonged intubation, extended ICU stay, and longer cardiopulmonary bypass duration were significantly associated with in-hospital mortality. **Conclusions:** Postoperative neurological complications were associated with prolonged ICU stay and increased in-hospital mortality. These findings emphasize the clinical importance of close neurological monitoring and perioperative strategies aimed at preserving cerebral perfusion and minimizing cardiopulmonary bypass duration in pediatric cardiac surgery.

## 1. Introduction

Congenital heart disease (CHD) is the most common congenital malformation worldwide, with an estimated prevalence of 8–12 cases per 1000 live births [1]. Advances in surgical techniques, cardiopulmonary bypass technology, and perioperative management have dramatically improved survival rates among children with CHD over the past decades [2]. However, with increased survival, attention has shifted toward long-term morbidities, particularly those affecting the central nervous system and neurodevelopmental outcomes [3].

Neurological complications remain among the most serious sequelae of pediatric cardiac surgery. These complications range from hypoxic–ischemic encephalopathy, ischemic stroke, seizures, and transient neurological deficits to more subtle but clinically significant cognitive, motor, and language impairments [4,5]. Such complications not only prolong hospital stay and increase healthcare utilization but also contribute substantially to long-term disability and reduced quality of life. Importantly, neonates and infants are at heightened risk due to the immaturity of the central nervous system, making them particularly vulnerable to hypoxia, ischemia, and reperfusion-related injury [6].

The etiology of neurological injury in this setting is multifactorial. Intraoperative factors such as the duration of cardiopulmonary bypass, degree of hypothermia, circulatory arrest strategies, and intraoperative hypoxia–reperfusion contribute significantly to cerebral vulnerability [7,8]. In addition, preoperative conditions—including chronic hypoxemia, impaired cerebral autoregulation, and preexisting neurological deficits—may interact with surgical stressors to worsen outcomes [9].

Despite decades of investigation, the reported incidence and factors associated with neurological complications in pediatric cardiac surgery remain inconsistent. Some studies have highlighted prolonged cardiopulmonary bypass time, deep hypothermic circulatory arrest, and complex surgical procedures as factors associated with neurological complications whereas others have questioned the strength of these associations [10,11,12]. Moreover, most prior studies have been limited by small sample sizes, heterogeneous patient populations, or narrow focus on specific surgical techniques. As a result, there remains a paucity of robust data regarding the true incidence and factors associated with neurological morbidity in pediatric patients undergoing diverse cardiac operations [13].

Given these uncertainties, comprehensive evaluation of neurological complications in large, heterogeneous cohorts is needed to inform perioperative strategies better and improve neurodevelopmental outcomes. Therefore, the present study aimed to determine the incidence of postoperative neurological complications in pediatric patients undergoing cardiac surgery at our institution, to identify perioperative and intraoperative factors associated with these complications, and to evaluate their impact on postoperative recovery and in-hospital outcomes. We hypothesized that prolonged cardiopulmonary bypass duration and perioperative clinical instability would be associated with a higher incidence of neurological complications and adverse postoperative outcomes.

## 2. Materials and Methods

The study protocol was reviewed and approved by the Medipol University Clinical Research Ethics Committee (Decision No: 923). All procedures were conducted in compliance with the ethical standards of the institutional and national research committees and adhered to the principles of the Declaration of Helsinki (1964) and its subsequent amendments. Given the retrospective design of the study, informed consent was not required; however, all patient data were anonymized and handled confidentially to protect privacy.

This was a retrospective, single-center observational study performed at Medipol University Hospital, a tertiary referral center for pediatric cardiovascular surgery. The study period extended from January 2021 to January 2022. Demographic, clinical, intraoperative, and postoperative variables relevant to neurological outcomes were retrospectively collected and analyzed from electronic medical records, operative notes, anesthesia charts, and intensive care unit (ICU) flow sheets. Two investigators independently reviewed the data, and discrepancies were resolved by consensus.

Surgical risk stratification was retrospectively performed using the Risk Adjustment for Congenital Heart Surgery (RACHS-1) classification system. Each surgical procedure was categorized into RACHS-1 risk groups (1–6) based on standardized definitions. Patients were further grouped into low-risk (RACHS 1–2), intermediate-risk (RACHS 3–4), and high-risk (RACHS 5–6) categories for descriptive and comparative analyses.

The study included patients younger than 18 years at the time of surgery who underwent either elective, or emergency cardiovascular surgery performed with cardiopulmonary bypass. Patients aged 18 years or older, those who died intraoperatively or before a neurological assessment could be completed, and those lacking postoperative neurological evaluation or neuroimaging when clinically indicated were excluded from the analysis.

Neurological complications were determined from clinical observations, neurology consultation records, and postoperative neuroimaging with computed tomography and/or magnetic resonance imaging when available. All patients underwent routine daily neurological screening during ICU stay by the attending intensivists including assessment of level of consciousness, motor response, pupillary reflexes, and seizure activity together with Glasgow Coma Scale assessment. Neurology consultation was requested in cases of abnormal neurological examination, unexplained delayed awakening, new-onset seizures, focal neurological deficits, or altered mental status. Cranial CT and/or MRI and electroencephalography (EEG) were not performed routinely in all patients but were obtained based on predefined clinical indications. Neuroimaging was specifically performed for suspected ischemic events, intracranial hemorrhage, hypoxic–ischemic injury, or persistent neurological abnormalities, while EEG was obtained when seizures were suspected or when altered consciousness persisted without clear structural findings on imaging. Neurological outcomes were ascertained throughout the postoperative hospital stay, including the immediate postoperative period, ICU course, and the remainder of the index hospitalization until discharge or in-hospital death.

Postoperative neurological complications were categorized as follows: seizures, defined as clinically observed convulsive episodes with electroencephalographic confirmation when available; ischemic stroke, defined as the presence of acute focal neurological deficits accompanied by neuroimaging evidence of cerebral infarction; intracranial hemorrhage, defined as parenchymal, subarachnoid, or intraventricular bleeding confirmed by computed tomography or magnetic resonance imaging; hypoxic–ischemic encephalopathy, defined by an encephalopathic clinical presentation (including altered mental status, coma, or seizures) supported by radiological findings consistent with hypoxic–ischemic brain injury.

### Statistical Analysis

All statistical analyses were conducted using IBM SPSS Statistics version 26.0 (IBM Corp., Armonk, NY, USA). Continuous variables were expressed as mean ± standard deviation (SD) for normally distributed data or as median with interquartile range (IQR) for non-normally distributed data. Categorical variables were expressed as frequencies and percentages. The Shapiro–Wilk test was used to evaluate the normality of continuous variables. Group comparisons were performed using Student’s *t*-test for normally distributed continuous variables and the Mann–Whitney U test for non-normally distributed variables. The Chi-square test or Fisher’s exact test was applied to compare categorical variables. For regression analyses, body surface area was dichotomized using the cohort median value (0.42 m^2^) as the cut-off point. Univariable logistic regression analysis was initially performed to explore associations with neurological complications. No multivariable models were constructed due to limited event numbers. A two-sided *p*-value < 0.05 was considered statistically significant.

The cut-off value for cardiopulmonary bypass duration (>133 min) was derived from the median value of the study cohort and was used for exploratory descriptive analyses. We acknowledge that dichotomizing continuous variables using median values may result in information loss and reduced statistical power; therefore, these analyses were considered exploratory.

## 3. Results

### 3.1. Patient Demographics and Baseline Characteristics

A total of 210 pediatric patients who underwent cardiovascular surgery during the study period were included in this retrospective analysis. Of these, 119 patients (56.7%) were male, and 91 patients (43.3%) were female, reflecting a slight predominance of males in this surgical cohort. The age distribution indicated that 117 patients (55.7%) were younger than 2 years, representing the largest age subgroup, while 32 neonates (15.2%) and 61 children aged 2–18 years (29%) comprised the remainder. Among the 20 patients with postoperative neurological complications, 6 (30.0%) were female, 1 (5.0%) was a neonate, 16 (80.0%) were younger than 2 years, and 4 (20.0%) died during hospitalization (Table 1).

### 3.2. Surgical Interventions

Analysis of the surgical procedures revealed a diverse range of congenital heart defect corrections. The most frequently performed operations included ventricular septal defect repair in 39 patients (18.6%), tetralogy of Fallot repair in 28 patients (13.3%), coarctation of the aorta repair in 24 patients (11.4%), and atrial septal defect closure in 13 patients (6.2%). Additional procedures, including Glenn shunt, Blalock–Taussig shunt, Fontan completion, pulmonary banding, and complex repairs such as Rastelli and Konno operations, unifocalization, atrioventricular septal defect repair were performed in smaller subsets. The cohort demonstrated a predominantly moderate-to-high surgical risk profile. The distribution of RACHS categories was as follows: 39.0% in RACHS 1–2 (low risk), 56.2% in RACHS 3–4 (moderate risk), and 4.8% in RACHS 5–6 (high risk). Although the proportion of patients in the highest risk categories (RACHS 5–6) was limited, the overall cohort was largely composed of moderate- and high-complexity surgical procedures.

### 3.3. Perioperative and Postoperative Course

During the postoperative period, 27 patients (12.9%) did not require inotropic support, whereas 94 patients (44.8%) required one or more inotropic agents, reflecting varying degrees of postoperative cardiac compromise.

Intraoperatively, 73 patients (54.9%) were managed with a pulsatile cardiopulmonary bypass flow strategy, and 128 patients (91.4%) received blood cardioplegia, highlighting contemporary perfusion and myocardial protection practices. The use of pulsatile cardiopulmonary bypass was not protocol-driven and was primarily based on individual surgeon preference rather than predefined clinical criteria. The incidence of postoperative neurological complications was 9.5% (95% CI: 6.2–14.3%), and in-hospital mortality was 8.6% (95% CI: 5.5–13.1%). Among the 20 patients who developed postoperative neurological complications, seizures were observed in 6 patients (30%), ischemic stroke or cerebral ischemia in 5 patients (25%), hypoxic–ischemic injury in 5 patients (25%), intracranial hemorrhage in 2 patients (10%), encephalopathy/cerebral edema in 1 patient (5%) and Guillain–Barré syndrome in 1 patient (5%) (Table 2).

Postoperative variables such as prolonged mechanical ventilation, extended ICU stay, and neurological complications were interpreted as markers of clinical severity rather than baseline factors associated with mortality.

### 3.4. Neurodiagnostic Work-Up

Cranial magnetic resonance imaging was performed in 12 patients (5.7%), and electroencephalography was obtained in 16 patients (7.6%). Postoperative neurological complications were identified at a median of 2 days (IQR: 1–3) following surgery among affected patients (*n* = 20).

### 3.5. Neurological Complications and Associated Outcomes

Patients were stratified according to the presence or absence of postoperative neurological complications, and demographic, perioperative, and postoperative characteristics were compared between groups. No statistically significant differences were observed with respect to sex, age, type of surgery, inotropic support requirement, use of pulsatile cardiopulmonary bypass, type of cardioplegia, cold agglutinin positivity, or overall, in-hospital mortality (*p* > 0.05).

However, the occurrence of postoperative neurological complications was significantly associated with prolonged postoperative mechanical ventilation and longer intensive care unit (ICU) stay. The median duration of postoperative intubation was 8.5 days [IQR: 2.5–13] in patients with neurological complications compared with 2 days [IQR: 1–4] in those without complications (*p* < 0.001). Similarly, the median ICU length of stay was significantly longer in patients with neurological complications (15 days [IQR: 6–29] vs. 4 days [IQR: 3–10]; *p* < 0.001), indicating a substantial increase in postoperative resource utilization associated with neurological events. Table 3 presents a comparative analysis of perioperative variables between patients with and without postoperative neurological complications.

### 3.6. Factors Associated with In-Hospital Mortality

Univariable logistic regression analysis was performed to explore factors associated with in-hospital mortality (Table 4). Variables significantly associated with increased in-hospital mortality included the presence of postoperative neurological complications (OR: 4.23; 95% CI: 1.34–13.38; *p* = 0.014), prolonged postoperative intubation (>2 days) (OR: 8.17; 95% CI: 1.83–36.50; *p* = 0.006), extended ICU stay (>5 days) (OR: 3.73; 95% CI: 1.18–11.73; *p* = 0.025), and cardiopulmonary bypass duration >133 min (OR: 6.78; 95% CI: 1.47–31.38; *p* = 0.014). These postoperative variables reflect downstream markers of clinical severity and postoperative course and were not intended for baseline risk stratification or predictive modeling at admission.

Conversely, a body surface area greater than 0.42 m^2^ was significantly associated with lower in-hospital mortality (OR: 0.14; 95% CI: 0.03–0.65; *p* = 0.012). These findings reflect statistical associations observed in univariable analyses and should be interpreted as descriptive rather than causal relationships.

## 4. Discussion

In this study, we evaluated the incidence of postoperative neurological complications in 210 pediatric patients who underwent cardiovascular surgery for CHD. The majority of patients, 117 (55.7%), were under 2 years of age, representing the most vulnerable age group. Postoperative neurological complications were observed in 20 patients (9.5%). Comparable findings have been reported in the literature. Abdshah et al. reported neurological complications in 14 children (5.2%), while Arslanoğlu et al., in a cohort of 3849 children, identified early neurological complications in 162 patients (4.2%), highlighting the association between early postoperative neurological events and increased mortality risk [4,7]. Desnous et al., in a study of 128 children with complex CHD, observed a higher incidence of perioperative clinical seizures, with neurological complications occurring in 10 patients (7.8%) [8]. The relatively higher complication rate in our study may reflect the complexity of the surgical procedures performed. In our cohort, 39 patients (18.6%) underwent ventricular septal defect repair, 28 patients (13.3%) underwent tetralogy of Fallot repair, 24 patients (11.4%) underwent coarctation repair, and 13 patients (6.2%) underwent atrial septal defect closure.

Prolonged ICU stay has been consistently identified as a significant risk factor for postoperative neurological complications, even after adjusting for variables such as cardiac lesion type, cardiopulmonary bypass duration, pre-existing brain injury, anesthetic exposure, and low regional cerebral oxygen saturation [9].

With neonatal survival rates following congenital heart surgery now exceeding 90%, quality of life and long-term neurodevelopmental outcomes have become critical clinical and research priorities. Between 25% and 90% of neonates who undergo cardiac surgery subsequently exhibit neurodevelopmental impairments. The emergence of this data over the past decade has prompted extensive investigation into the underlying mechanisms and potential therapeutic strategies, taking into account the complexity of cardiac lesions, surgical procedures, and genetic factors [9]. In our cohort, 41 patients (19.5%) had chronic comorbidities preoperatively, of whom 16 patients already had documented neurological disorders. Genetic syndromes included Down syndrome in 14 patients, trisomy 18 in 1 patient, Noonan syndrome in 1 patient, Zellweger syndrome in 1 patient, and Williams syndrome in 2 patients. Additionally, 6 patients had epilepsy. Previous studies indicate that at least 20–30% of pediatric CHD patients present with an associated genetic syndrome or diagnosis [10].

Neuroimaging studies in term neonates with complex CHD have revealed preoperative brain injury in 15–40% of cases, predominantly affecting white matter. Neurological events in these patients, such as seizures and strokes, are associated with long-term deficits in cognitive, motor, language, and socio-emotional functioning, as well as increased risk for attentional difficulties. Factors such as the severity of cardiac disease, age at surgery, and length of hospital stay further contribute to long-term neurodevelopmental risk [2].

Intraoperative cerebral hypoperfusion remains a key mechanism contributing to postoperative neurological complications. Recent attention has focused on monitoring regional cerebral oxygen saturation using near-infrared spectroscopy, a non-invasive method providing real-time assessment of cerebral perfusion during surgery [11]. Despite the development of algorithms to prevent near-infrared spectroscopy-detected cerebral desaturation, the relationship between intraoperative regional cerebral oxygen saturation and postoperative neurological outcomes remains inconclusive [12]. Emerging evidence, however, suggests that low regional cerebral oxygen saturation during cardiopulmonary bypass is associated with adverse neurodevelopmental outcomes at 1–2 years postoperatively [13,14]. In our study, although patients were monitored with near-infrared spectroscopy, the retrospective design and incomplete documentation precluded analysis of the relationship between regional cerebral oxygen saturation and postoperative neurological complications.

In our cohort, 18 patients (8.6%) died during hospitalization. Although neurological complications were the primary focus of this study, univariable logistic regression analysis showed that postoperative neurological complications, prolonged intubation (>2 days), prolonged ICU stay (>5 days), and cardiopulmonary bypass duration >133 min were significantly associated with in-hospital mortality. These findings highlight the close association between neurological complications and adverse postoperative outcomes rather than introducing a mortality prediction model. Postoperative variables such as ventilation duration, ICU stay, and neurological complications mainly reflect clinical severity and postoperative course. Accordingly, our findings should be interpreted as descriptive associations rather than causal relationships. Importantly, prolonged intubation and extended ICU stay should be interpreted as downstream indicators of clinical severity rather than variables suitable for early risk stratification or admission-based prognostic assessment.

Prolonged ICU stay and mechanical ventilation are well-established factors associated with mortality following pediatric cardiac surgery. Hein et al. reported that prolonged mechanical ventilation and extended ICU stay increased mortality, with 36% of cardiac surgery patients requiring >14 days of ICU care due to non-cardiac organ failure [15]. Similarly, Fernandez-Zamora et al. estimated that prolonged ICU stay, and mechanical ventilation contributed to mortality in 20% of patients, primarily due to multi-organ failure and sepsis [16]. Strategies aimed at early extubation, sedation protocols, prompt detection of hemodynamic instability, and prevention of infection and other complications are therefore critical for improving neurodevelopmental outcomes [17].

Regarding myocardial protection, blood cardioplegia was used in 128 patients (91.4%), and Del Nido cardioplegia in 12 patients (8.6%), with no statistically significant difference in the incidence of postoperative neurological complications. Various cardioplegia solutions have been developed to target intracellular mechanisms and preserve myocardial function without causing major metabolic disturbances, yet consensus on the optimal cardioplegia strategy remains lacking [18].

Similarly, the use of pulsatile cardiopulmonary bypass in our cohort was not associated with a reduction in neurological complications. Patel et al. reported that although pulsatile cardiopulmonary bypass improved the physiological pulsatility index in both acyanotic and cyanotic patients, no significant differences in cerebral hemodynamics or clinical outcomes were observed, consistent with our findings [19].

Finally, cardiopulmonary bypass duration > 133 min was significantly associated with in-hospital mortality in our cohort. Consistent with our findings, Akingeneye et al. reported that prolonged cardiopulmonary bypass duration was associated with increased postoperative morbidity and mortality, as well as prolonged mechanical ventilation and longer hospital stay in pediatric cardiac surgery patients. These findings further support the close relationship between extended cardiopulmonary bypass exposure and adverse postoperative outcomes [20].

Due to the retrospective design and the limited number of neurological and mortality events, multivariable regression analysis could not be reliably performed without a substantial risk of model overfitting. Therefore, the present findings should be interpreted as statistical associations rather than independent predictors or causal relationships. Larger prospective studies are warranted to confirm these associations and to better characterize factors associated with neurological morbidity and mortality following pediatric cardiac surgery.

## 5. Conclusions

Postoperative neurological complications following pediatric cardiovascular surgery represent a significant source of morbidity and mortality. Close intraoperative and postoperative neurological monitoring of high-risk patients, implementation of strategies to preserve cerebral perfusion during surgery, and minimizing cardiopulmonary bypass duration whenever feasible may be important components of perioperative management. The potential role of advanced monitoring techniques, such as near-infrared spectroscopy, should be further evaluated in future prospective studies.

### Limitations

The primary limitations of this study include its retrospective single-center design and the relatively small number of neurological and mortality events, which precluded reliable multivariable regression modeling and increased the risk of model overfitting. Consequently, the results should be interpreted as descriptive associations rather than causal inferences. In addition, neurological imaging and electroencephalography were performed based on clinical indication rather than systematically in all patients, which may have introduced detection bias and potential under-ascertainment of subclinical neurological events. The retrospective nature of data collection further limits control over diagnostic timing and completeness. Long-term neurodevelopmental outcomes could not be evaluated. Finally, residual confounding related to unmeasured perioperative variables cannot be excluded.

## Figures and Tables

**Table 1:** Baseline demographic characteristics of the study population (*n* = 210).

Variable	Total Cohort (*n* = 210)	Neurological Complications (*n* = 20)
Male sex, *n* (%)	119 (56.7)	14 (70.0)
Female sex, *n* (%)	91 (43.3)	6 (30.0)
Neonates, *n* (%)	32 (15.2)	1 (5.0)
Age < 2 years, *n* (%)	117 (55.7)	16 (80.0)
Age 2–18 years, *n* (%)	61 (29.0)	4 (20.0)
In-hospital mortality, *n* (%)	18 (8.6)	4 (20.0)

**Table 2:** Types of Postoperative Neurological Complications.

Type of Neurological Injury	*n* (%)
Seizures	6 (30%)
Ischemic stroke/cerebral ischemia	5 (25%)
Hypoxic–ischemic injury	5 (25%)
Intracranial hemorrhage	2 (10%)
Encephalopathy/cerebral edema	1 (5%)
Guillain–Barré syndrome	1 (5%)

**Table 3:** Perioperative and early postoperative characteristics in patients with and without neurological complications.

	Neurological Complications	No Neurological Complications	
	Median [IQR]	Median [IQR]	*p*
Body Mass Index	14.35 [12.7–15.3]	14.4 [13.1–16.9]	0.275
Body Surface Area	0.39 [0.31–0.42]	0.44 [0.29–0.71]	0.232
Postoperative intubation time	8.5 [2.5–13]	2 [1–4]	**<0.001**
ICU stay time (day)	15 [6–29]	4 [3–10]	**<0.001**
Cross Clamp time (min)	67 [53–112]	91 [60–126]	0.367
Cardiopulmonary bypass time (min)	136 [95–214]	130.5 [93.5–171]	0.584

Values are presented as median [IQR]. *p* values obtained using Mann–Whitney U test.

**Table 4:** Univariable logistic regression analysis for factors associated with in-hospital mortality.

	Univariable LR	
	OR (95% CI)	*p*
Sex (Male)	1.22 (0.45–3.29)	0.691
Age ref = Newborn	1	0.237
<2 years old	0.51 (0.16–1.60)	0.245
>2 years old	0.28 (0.06–1.26)	0.096
Body Mass Index > 14.5	0.38 (0.11–1.25)	0.109
Body Surface Area > 0.42	0.14 (0.03–0.65)	**0.012**
Neurological Complications	4.23 (1.34–13.38)	**0.014**
Inotrope requirement	0.56 (0.05–6.48)	0.647
Pulsatile flow model	0.25 (0.05–1.31)	0.101
Chronic disease	2.96 (1.07–8.18)	**0.037**
Cold agglutination positivity	1.07 (0.13–8.87)	0.950
Intubation time > 2 days	8.17 (1.83–36.50)	**0.006**
ICU stay time > 5 days	3.73 (1.18–11.73)	**0.025**
Cross Clamp time (min) > 89.5	2.13 (0.61–7.43)	0.236
Cardiopulmonary bypass time > 133 min	6.78 (1.47–31.38)	**0.014**

LR: Logistic regression, OR: Odds ratio, CI: Confidence interval.

## Data Availability

The data presented in this study are available from the corresponding author upon reasonable request. The data are not publicly available due to ethical restrictions and patient confidentiality.

## Abbreviations

The following abbreviations are used in this manuscript:

CHD	Congenital heart disease
ICU	Intensive care unit
RACHS	Risk Adjustment for Congenital Heart Surgery

## References

[1] Liu Y., Chen S., Zühlke L., Babu-Narayan S.V., Black G.C., Choy M.-K., Li N., Keavney B.D. (2020). Global prevalence of congenital heart disease in school-age children: A meta-analysis and systematic review. BMC Cardiovasc. Disord..

[2] Butler S.C., Sadhwani A., Rofeberg V., Cassidy A.R., Singer J., Calderon J., Wypij D., Newburger J.W., Rollins C.K. (2022). Neurological features in infants with congenital heart disease. Dev. Med. Child. Neurol..

[3] Howell H.B., Zaccario M., Kazmi S.H., Desai P., Sklamberg F.E., Mally P. (2019). Neurodevelopmental outcomes of children with congenital heart disease: A review. Curr. Probl. Pediatr. Adolesc. Health Care.

[4] Abdshah A., Mirzaaghayan M., Heidari M., Memarian S., Amanollahi M., Nazeri A., Gharib B. (2023). Incidence of neurological complications following pediatric heart surgery and its association with neutrophil-to-lymphocyte ratio. Health Sci. Rep..

[5] Bamaga A., Bahafzalla R., Alamoudi N., Alqarni S., Alotaibi R., Almontashri A., Faisal Z. (2025). Evaluating neurological outcomes in children undergoing cardiac surgery in the Middle East: A retrospective study. Pediatr. Med..

[6] Raffa G.M., Agnello F., Occhipinti G., Miraglia R., Re V.L., Marrone G., Tuzzolino F., Arcadipane A., Pilato M., Luca A. (2019). Neurological complications after cardiac surgery: A retrospective case-control study of risk factors and outcome. J. Cardiothorac. Surg..

[7] Arslanoğlu E., Kara K.A., Yiğit F., Arkan C., Uslu U., Şavluk Ö.F., Yılmaz A.A., Tunçer E., Çine N., Ceyran H. (2021). Neurological complications after pediatric cardiac surgery. Cardiothorac. Surg..

[8] Desnous B., Lenoir M., Doussau A., Marandyuk B., Beaulieu-Genest L., Poirier N., Carmant L., Birca A. (2019). Epilepsy and seizures in children with congenital heart disease: A prospective study. Seizure.

[9] Andropoulos D.B., Easley R.B., Gottlieb E.A., Brady K. (2019). Neurologic Injury in Neonates Undergoing Cardiac Surgery. Clin. Perinatol..

[10] Ko J.M. (2015). Genetic Syndromes associated with Congenital Heart Disease. Korean Circ. J..

[11] Denault A., Deschamps A., Murkin J.M. (2007). A proposed algorithm for the intraoperative use of cerebral near-infrared spectroscopy. Semin. Cardiothorac. Vasc. Anesth..

[12] Semrau J.S., Motamed M., Ross-White A., Boyd J.G. (2021). Cerebral oximetry and preventing neurological complication post-cardiac surgery: A systematic review. Eur. J. Cardiothorac. Surg..

[13] Aly S.A., Zurakowski D., Glass P., Skurow-Todd K., Jonas R.A., Donofrio M.T. (2017). Cerebral tissue oxygenation index and lactate at 24 hours postoperative predict survival and neurodevelopmental outcome after neonatal cardiac surgery. Congenit. Heart Dis..

[14] Kussman B.D., Wypij D., Laussen P.C., Soul J.S., Bellinger D.C., DiNardo J.A., Robertson R., Pigula F.A., Jonas R.A., Newburger J.W. (2010). Relationship of intraoperative cerebral oxygen saturation to neurodevelopmental outcome and brain magnetic resonance imaging at 1 year of age in infants undergoing biventricular repair. Circulation.

[15] Hein O.V., Birnbaum J., Wernecke K.D., Konertz W., Spies C. (2006). Intensive care unit stay of more than 14 days after cardiac surgery is associated with non-cardiac organ failure. J. Int. Med. Res..

[16] Fernandez-Zamora M.D., Gordillo-Brenes A., Banderas-Bravo E., Arboleda-Sánchez J.A., Hinojosa-Pérez R., Aguilar-Alonso E. (2018). Prolonged Mechanical Ventilation as a Predictor of Mortality After Cardiac Surgery. Respir. Care.

[17] Peyvandi S., Latal B., Miller S.P., McQuillen P.S. (2019). The neonatal brain in critical congenital heart disease: Insights and future directions. Neuroimage.

[18] Mohammed S., Menon S., Gadhinglajkar S.V., Baruah S.D., Ramanan S.V., Gopalakrishnan K.A., Suneel P.R., Dharan B.S. (2022). Clinical outcomes of del nido cardioplegia and st thomas blood cardioplegia in neonatal congenital heart surgery. Ann. Card. Anaesth..

[19] Patel K., Lin T.K., Clark J.B., Ceneviva G.D., Imundo J.R., Spear D., Kunselman A.R., Thomas N.J., Myers J.L., Ündar A. (2025). Randomized Trial of Pulsatile and Nonpulsatile Flow in Cyanotic and Acyanotic Congenital Heart Surgery. World J. Pediatr. Congenit. Heart Surg..

[20] Akingeneye P., Bradley D.J., Mucumbitsi J., Nyilimanzi N., Mutabandama Y., Rusingiza E.K. (2025). Clinical outcomes of children operated for congenital heart diseases in Rwanda: A 14-year retrospective analysis. Int. J. Cardiol. Congenit. Heart Dis..

